# A multi-region assessment of population rates of cardiac catheterization and yield of high-risk coronary artery disease

**DOI:** 10.1186/1472-6963-11-323

**Published:** 2011-11-24

**Authors:** Fiona M Clement, Braden J Manns, Brenda Brownell, Peter D Faris, Michelle M Graham, Karin Humphries, Michael Love, Merril L Knudtson, William A Ghali

**Affiliations:** 1 Department of Medicine, Faculty of Medicine, University of Calgary, Foothills Medical Centre - North Tower, 9th Floor, 1403 - 29th Street NW, Calgary, AB T2N 2T9, Canada; 2 Department of Community Health Sciences, Faculty of Medicine, University of Calgary, TRW Building 3rd Floor, 3280 Hospital Drive NW, Calgary, Alberta T2N 4Z6, Canada; 3 Centre for Health and Policy Studies, Faculty of Medicine, University of Calgary, TRW Building 3rd Floor, 3280 Hospital Drive NW, Calgary, Alberta T2N 4Z6, Canada; 4 Department of Medicine, Dalhousie University, Room 442, Bethune Building, VG Site, 1276 South Park Street, Halifax, Nova Scotia, B3H 2Y9, Canada; 5 Capital Health, 1278 Tower Rd, Halifax, Nova Scotia, B3H 2Y9, Canada; 6 Department of Medicine, University of Alberta, 13-103 Clinical Sciences Building, 11350 - 83 Avenue, Edmonton, Alberta, T6G 2G3, Canada; 7 Division of Cardiology, University of British Columbia, 2775 Laurel St, 9th Floor, Vancouver, British Columbia V5Z 1M9, Canada; 8 Centre for Health Evaluation and Outcome Sciences, St. Paul's Hospital, 620B - 1081 Burrard Street, Vancouver, British Columbia, V6Z 1Y6, Canada

## Abstract

**Background:**

There is variation in cardiac catheterization utilization across jurisdictions. Previous work from Alberta, Canada, showed no evidence of a plateau in the yield of high-risk disease at cardiac catheterization rates as high as 600 per 100,000 population suggesting that the optimal rate is higher. This work aims 1) To determine if a previously demonstrated linear relationship between the yield of high-risk coronary disease and cardiac catheterization rates persists with contemporary data and 2) to explore whether the linear relationship exists in other jurisdictions.

**Methods:**

Detailed clinical information on all patients undergoing cardiac catheterization in 3 Canadian provinces was available through the Alberta Provincial Project for Outcomes Assessment in Coronary Heart (APPROACH) disease and partner initiatives in British Columbia and Nova Scotia. Population rates of catheterization and high-risk coronary disease detection for each health region in these three provinces, and age-adjusted rates produced using direct standardization. A mixed effects regression analysis was performed to assess the relationship between catheterization rate and high-risk coronary disease detection.

**Results:**

In the contemporary Alberta data, we found a linear relationship between the population catheterization rate and the high-risk yield. Although the yield was slightly less in time period 2 (2002-2006) than in time period 1(1995-2001), there was no statistical evidence of a plateau. The linear relationship between catheterization rate and high-risk yield was similarly demonstrated in British Columbia and Nova Scotia and appears to extend, without a plateau in yield, to rates over 800 procedures per 100,000 population.

**Conclusions:**

Our study demonstrates a consistent finding, over time and across jurisdictions, of linearly increasing detection of high-risk CAD as population rates of cardiac catheterization increase. This internationally-relevant finding can inform country-level planning of invasive cardiac care services.

## Background

Cardiac catheterization is one of the most commonly performed invasive diagnostic procedures, yet it is also a procedure for which there is tremendous variation in utilization across countries and across jurisdictions within countries [1-5]. This variation exists in the context of a relative void of knowledge around what the optimal rate of cardiac catheterization should really be. Developed countries with relatively low rates of cardiac catheterization, such as the United Kingdom, may be under-using the procedure, or alternatively, countries with high population rates, such as the United States, may be overusing the procedure [2,6].

A previous study from our group provided some insight into where a theoretically 'optimal' rate of cardiac catheterization may reside [7]. Using 1995 to 2002 cardiac catheterization data from multiple health regions in Alberta, Canada, we reported that the yield of detection of high-risk coronary artery disease (CAD) (i.e. triple vessel disease, left main disease, and two vessel disease with proximal LAD involvement) appeared to increase linearly with an increase in cardiac catheterization rates within health regions [7]. Furthermore, there was no evidence of a plateau in the yield of high-risk disease at cardiac catheterization rates as high as 600 procedures per 100,000 population, suggesting that the optimal catheterization rate to detect high-risk CAD (with continuing returns of high-risk disease detection) is higher than this level. The detection of high-risk disease is indeed one of the primary goals of cardiac catheterization, as only patients with high risk disease have been shown to have significant survival and quality of life benefits associated with the use of revascularization, particularly in high-risk disease sub-groups [8,9].

Given that catheterization rates have continued to change over time and that significant cardiac catheterization rate variation persists across geographic areas, we conducted this ecological study to further evaluate the relationship between population rates of cardiac catheterization and the yield of high-risk disease within different health care regions. We compiled detailed cardiac catheterization data to determine if the previously demonstrated linear increase in yield of high-risk disease seen in Alberta, Canada persists in more recent years as catheterization rates have continued to rise in that province. We also explored whether this relationship exists in health care regions in two other Canadian provincial jurisdictions, British Columbia and Nova Scotia.

## Methods

### Data Sources

We used the Alberta Provincial Project for Outcomes Assessment in Coronary Heart (APPROACH) disease database, which is a geographically defined, population-based registry that captures all patients undergoing cardiac catheterization in Alberta [10]. Corresponding cardiac catheterization data compiled through the partner cardiac registry initiatives in British Columbia and Nova Scotia were similarly obtained. The databases from each of the three Canadian provinces contain detailed clinical information, including demographic characteristics, co-morbidities and therapeutic interventions. Information on coronary anatomy is stored using a cardiac reconstruction software program (Heartview, Siemens Medical Systems). For the purposes of this analysis, high-risk CAD is defined as greater than 50% stenosis of the left-main coronary artery, or similar stenotic disease involving 3-vessels, or 2-vessels disease with involvement of the proximal left anterior descending artery [7]. Ethics approval was obtained from each respective academic institution ethics review board.

### Study Regions and Time Periods

Alberta APPROACH data from Jan 1, 1995 to Dec 31, 2006 were used. Repeat procedures were excluded so that individual patients would only be included once in the analysis. The previous work reported data from 1995 to 2001 ("time period 1") [7]. Due to a health region boundary change in April, 2002, the data for time period 1 (1995-2001) were re-analyzed for this study using the Alberta lay-out of 9 health regions that existed in 2006 to allow for seamless comparison in yield of high-risk disease across the two time periods. Only 2 of Alberta's 9 health regions have cardiac catheterization and revascularization facilities. These regions provide invasive investigation and revascularization for the entire province.

The Nova Scotia and British Columbia cardiac registry initiatives provided corresponding data from 2002-2004 and 2000-2005, respectively. This represents the entire dataset within each province for which Heartview and catheterization data are available. The dataset for each province includes all health authorities within each province (i.e. complete geographically inclusive capture). Nova Scotia has 9 District Health Authorities with cardiac catheterization provided by 1 centre. British Columbia has 5 Health Authorities, with cardiac catheterization services offered in three urban centres.

### Analysis

Analyses were completed for males and females separately as the prevalence of coronary artery disease differs between males and females and because the optimal catheterization rate may differ between sexes. Patients were categorized into health regions based on their residential postal code. All catheterization procedures were included. For each region, the population rates of catheterization and high-risk detection were calculated. Cardiac catheterization rates were derived as the total number of catheterization procedures divided by the total population over the age of 20. Rates of high-risk CAD detected were calculated as the number of patients with high-risk CAD detected divided by the total population over the age of 20. Subsequently, using direct standardization, age-adjusted rates were calculated. Data were categorized into 5 age categories; 20-34, 35-49, 50-64, 65-74 and over 75. The number of cardiac catheterizations within each age group was divided by the population in that category. Rates of high-risk CAD were calculated in the same manner. Weighting the age-specific rates with the 1996 Canadian population, we thus obtained age-adjusted cardiac catheterization and high-risk detection rates per 100,000 population. The 1996 census was used as the population reference standard for age adjustment (by direct standardization) because this standard has been used in other notable procedure rate work [11], and comparability of rates across studies was desired.

Subsequent analyses used these adjusted catheterization rates and high-risk detection rates as data points. Initially, a scatter plot of the catheterization rate versus the high-risk detection rate was constructed, with each data point representing an individual year of data for a particular region. For the Alberta data, we compared more recent years of data with earlier years, in order to determine whether relationships persisted over time. For the other two provinces, the entire time period available was reported without distinction between years.

Given that the unit of analysis for this study is the health care region, rather than the individual patient, we employed a least squares linear regression analysis to assess the relationship between catheterization rate and high-risk CAD detection. As the observations from each region are not independent, we performed hierarchical modeling using a mixed effects linear model with a random effect for each region. In this analysis, we modeled a shared fixed intercept (fixed at zero) and random slopes. The results of this model were then used to plot a single weighted line to reflect the linear relationship between catheterization rate and high-risk CAD detection rate for all regions. The line of "best fit" with 95% confidence lines was then overlaid on the scatter plot of observed data. To explore if the relationship might be different for patients with acute coronary syndrome (ACS) or for those with stable coronary disease, we repeated the above analysis, stratified by indication.

Lastly, we tested quadratic terms in the modeling process for the data from each of the provinces studied. A statistically significant quadratic term would indicate evidence of a plateau in the yield of high-risk CAD associated with increased catheterization rates. This was performed separately for both time periods studied in the Alberta data, and repeated for Nova Scotia and British Columbia data.

Recognizing the complexity of the mixed effects modeling described above, we performed a sensitivity analysis using simpler ordinary least squares regression. This latter analysis, while simpler, overlooks the non-independence of some of the data points analyzed. The findings of this sensitivity analysis are not presented here but were similar to those of our hierarchical mixed effects analysis.

## Results

### Alberta: Time Period 1 vs. Time Period 2

Table 1 presents the patient characteristics, intervention rates and outcomes by regional tertile of utilization in time period 1 and time period 2. The clinical characteristics and utilization are generally stable across tertiles and time periods. Table 2 shows the average population of males and females over 20 years of age, along with the crude and adjusted cardiac catheterization rates per 100,000 for each health region in Alberta in time periods 1 and 2. For males, the adjusted catheterization rates ranged from 379.7 to 538.4 per 100,000 in time period 1. In time period 2, the range is higher with slightly less variation -- i.e. population rates ranging from 438.9 to 587.8 per 100,000. There is an increase between time periods for both sexes, though females continued to have consistently lower rates than males.

**Table 1:** Alberta patient characteristics by tertile of catheterization rate for time period 1 and time period 2

	Lowest (Regions 1,2,5)		Middle (Regions 6,8,9)		Highest (Regions 3,4,7)	
**Characteristic (%)**	**Time period 1 (N = 5062)**	**Time period 2 (N = 5994)**	**Time period 1 (N = 16154)**	**Time period 2 (N = 19508)**	**Time period 1 (N = 23593)**	**Time period 2 (N = 25422)**

Mean Age (yrs.)	63.5	63.8	62.5	62.2	62.4	62.1
Male	69.4	69.6	71.3	70.3	68.5	70.1
Current Smoker	24.6	25.8	27.5	29.6	25.6	26.2
Cerebrovascular disease	6.7	7.1	6.2	6.4	6.7	6.8
Congestive Heart Failure	14.4	14.6	15.5	14.7	14.3	14.2
COPD	10.8	16.9	9.2	13.1	12.3	18.4
Diabetes	19.3	24.0	20.1	24.4	18.8	22.6
Dialysis	1.2	1.2	1.8	1.8	1.5	1.4
Hyperlipidemia	50.1	76.1	48.7	72.0	48.3	72.5
Hypertension	52.7	66.7	52.0	64.7	52.7	64.2
Lytic	10.7	9.4	7.1	5.0	7.9	3.0
Liver/GI	3.7	8.3	2.9	6.1	4.5	8.7
Malignancy	4.1	4.4	2.9	3.0	4.4	4.7
Peripheral Vascular disease	8.1	7.4	6.3	7.6	8.6	7.4
Previous CABG	6.4	3.4	7.5	3.0	6.4	3.1
Previous PCI	8.2	4.4	6.1	3.4	9.7	4.5
Indication for catheterization						
Stable angina	26.4	27.6	25.5	30.5	29.7	30.0
Unstable angina	30.3	22.1	29.2	18.1	29.6	21.2
Myocardial infarct.	30.0	37.4	28.5	37.2	27.3	35.9
Other	13.3	12.9	16.8	14.2	13.4	12.9
Coronary Anatomy						
Normal	20.3	25.9	21.2	26.7	25.9	29.7
Low risk	39.6	39.2	37.0	37.1	38.7	38.9
2-vessel involving PLAD	4.2	3.4	5.2	3.4	3.8	2.8
3-vessel	27.9	23.3	27.8	23.7	24.7	21.6
Left Main	8.0	7.3	8.8	7.0	7.0	6.4
Revasc. within 1 year	54.0	54.5	51.8	55.0	50.3	51.0
CABG within 1 year	22.9	16.7	21.9	17.2	18.8	14.8
PCI within 1 year	32.5	38.7	31.5	39.1	33.0	37.4
Death within 1 year	4.9	3.9	5.7	4.6	4.6	4.2

**Table 2:** Average population and average cardiac catheterization rates per 100 000 population over 20 years of age in Alberta health regions

	Time period 1 (1995-2001)						Time period 2 (2002-2006)					
**Health Region**	Population aged > 20 yrs*		**Crude catheterization rate**		Adjusted catheterization rate**		Population aged > 20 yrs*		**Crude catheterization rate**		Adjusted catheterization rate**	

	**Male**	**Female**	**Male**	**Female**	**Male**	**Female**	**Male**	**Female**	**Male**	**Female**	**Male**	**Female**

1	49015	51643	462.1	191.4	442.3	183.8	51776	54544	475.6	207.4	438.9	196.5
2	31479	32202	432.6	185.3	430.7	183.6	35187	35360	514.0	247.6	515.4	246.2
3	352016	362599	466.6	214.9	538.4	257.0	410790	421669	477.0	204.6	540.4	242.6
4	89896	92029	506.0	218.8	502.7	225.5	99557	101748	547.0	251.3	536.2	255.5
5	36905	38073	438.4	183.4	379.7	162.4	38420	39702	602.9	249.2	512.6	221.7
6	317698	332086	463.3	181.7	494.6	200.3	351822	366323	505.6	217.8	526.8	235.2
7	56644	55758	480.2	190.8	483.6	215.2	59643	58513	544.5	243.5	534.0	270.6
8	41519	39831	384.9	141.8	432.4	177.9	45536	43794	532.0	257.2	587.8	310.6
9	18810	17442	309.0	102.4	474.3	188.0	23879	21521	361.6	150.7	527.3	293.0

* Based on the average of populations within time period

** Adjusted for age

**Figure 1:**
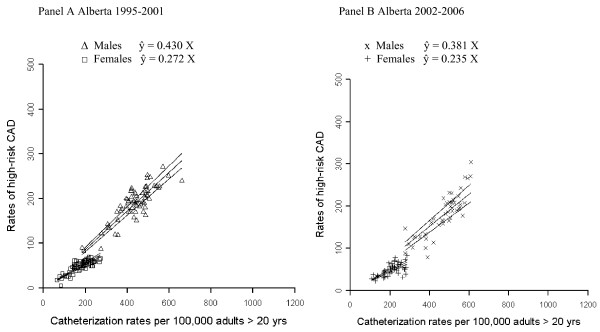
**Random slopes regression lines with 95% confidence bands for Alberta in time period 1 (Panel A - 1995-2001) and time period 2 (Panel B - 2002-2006)**.

The scatter plot of catheterization rates vs. high-risk rates and the results of the hierarchical modeling are presented in Figure 1. Each individual point represents one year of data for one health region. The results for 9 health regions analyzed in time period 1 (Panel A) confirm our previously reported findings for that period [7]. Time period 2 (Panel B), meanwhile, continues to show a positive association between cases of high-risk CAD detected and catheterization rates.

Describing the relationship quantitatively, for males in time periods 1 and 2, for every 2.3 and 2.6 catheterization procedures performed, respectively, an additional high-risk CAD patient was detected. For females, the detection rate is lower than males with an additional high-risk patient detected in every 3.7 and 4.3 catheterization procedures performed in time periods 1 and 2, respectively.

### British Columbia and Nova Scotia

Table 3 presents the population, crude catheterization rate and adjusted catheterization rate for British Columbia and Nova Scotia. In general, the rates in both British Columbia and Nova Scotia are higher than those seen in Alberta, and as in Alberta, males have consistently higher rates than females.

**Table 3:** Average population and average cardiac catheterization rates per 100 000 population over 20 years of age in British Columbia Health Authorities (2000-2005) and Nova Scotia District Health Authorities (2002-2004)

Health Authority	Population aged > 20 yrs*		Crude catheterization rate		Adjusted catheterization rate**	
**British Columbia**	**Male**	**Female**	**Male**	**Female**	**Male**	**Female**

1	272083	284886	589.9	279.1	465.5	243.0
2	279301	299197	804.8	358.4	644.5	301.3
3	424195	445112	510.4	214.1	507.7	222.0
4	546130	565514	686.5	313.8	673.4	315.2
5	112268	105168	465.1	219.4	484.9	259.8

**Nova Scotia**						
1	23361	24312	779.2	446.2	617.8	352.7
2	23839	25080	729.8	386.8	615.2	334.7
3	30218	32002	650.3	350.1	545.9	305.3
4	26439	27537	887.2	468.4	790.1	424.6
5	12441	13209	888.2	575.5	697.7	443.1
6	17421	18840	617.1	291.9	524.9	249.8
7	17349	17959	582.1	314.7	476.8	267.0
8	47055	52323	1048.7	488.3	856.9	408.9
9	145278	156187	766.2	416.5	778.7	427.6

* Based on the average of populations within time period

** Adjusted for age

**Figure 2:**
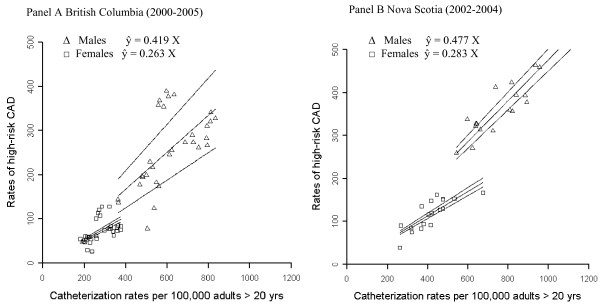
**Random slopes regression lines with 95% confidence bands for British Columbia (2000-2005, Panel A) and Nova Scotia (2002-2004, Panel B)**.

The linear relationship between catheterization rate and high-risk yield is similarly demonstrated in both provinces, with findings that closely resemble those from Alberta (Figure 2). In British Columbia, every 2.4 catheterizations results in an additional high-risk case for males whereas for females, every 3.8 catheterizations results in an additional high-risk case. The detection rate is slightly higher in Nova Scotia with an additional high-risk case in every 2.1 catheterizations for males and every 3.5 procedures for females.

### Formal Testing for a Plateau in Yield of High-risk Disease

We formally assessed whether a plateau was evident in the high-risk CAD detection rate as catheterization rates increased. When using the Alberta data across both time points, the coefficient of the quadratic term entered into the regression model was not significant (p-value = 0.64). Similarly, there was no evidence of a significant quadratic term in either British Columbia (p-value = 0.67) or Nova Scotia (p-value = 0.61). This finding indicates that there is no statistical evidence of a plateau in yield of high-risk disease, in a range of data points extending to the highest catheterization rates seen in Nova Scotia of 850 procedures per 100,000 population.

### Stratified Analysis by Indication for Catheterization

**Figure 3:**
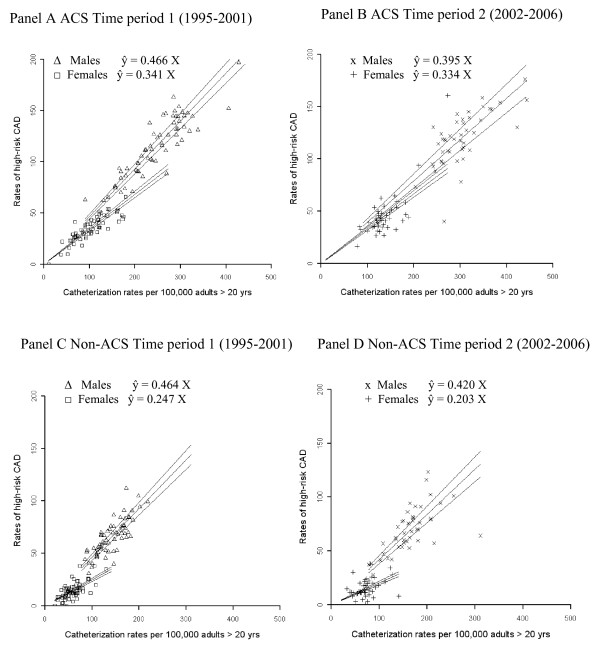
**Random slopes regression lines for Alberta stratified by indication for catheterization (ACS/Non-ACS) in time period 1 and time period 2**.

A similar linear relationship was found for both ACS and non-ACS subgroups, and on formal testing for evidence of a plateau, there was again no evidence of a statistically significant plateau in the relationship between population rates of catheterization and yield of high risk disease (test for quadratic term indicating plateau: p-value = 0.43 for ACS and p-value = 0.167 for non-ACS) (Figure 3). As observed in the overall analysis, the yield of high-risk cases is lower in females than males for both subgroups and both time periods.

## Discussion

Our study demonstrates a consistent finding, over time and across jurisdictions, of linearly increasing detection of high-risk CAD as population rates of cardiac catheterization increase. The results presented here extend our previously reported findings [7], by demonstrating the consistency of this finding across provinces. In addition, the linear increase in detection of high-risk disease appears to extend to catheterization rates beyond 800 procedures per 100,000 population.

These findings from Canada are of international importance, because they provide insights for countries with both lower and higher population rates of cardiac catheterization. For countries with lower rates than Canada's, such as the United Kingdom (catheterization rate for 2009: 380 per 100,000 [12]), Greece (catheterization rate for 2007: 308 per 100,000 [13]), Spain (catheterization rate for 2007: 282 per 100,000 [13]) and Poland (catheterization rate for 2007: 337 per 100,000 [13]), our findings would suggest a high likelihood of linearly increasing yield of high-risk disease if more procedures are performed, assuming that the burden of disease at a population-level is comparable to Canada's, as would be expected across many Westernized developed countries [6]. Meanwhile, for countries with higher catheterization rates, such as the United States (catheterization rate for 1995/96 for Medicare enrollees: 2270 per 100,000 [14]), Germany (catheterization rate for 2007: 1229 per 100,000 [13])and Belgium (catheterization rate for 2007: 713 per 100,000 [13]), our findings provide a methodological template for similar evaluations of diagnostic yield [2]. Indeed, a similar statistical analysis, with exploration of the presence or absence of a plateau in yield of high-risk disease beyond population rates of 1000 catheterization procedures per 100,000 population would be very informative for such a country.

Implicit to our study is the assertion that the detection of high-risk coronary artery disease is clinically important. In the presence of coronary disease affecting the left main coronary artery, or the three main coronary vessels, previous randomized trials confirm survival benefit from revascularization with either coronary artery bypass grafting (CABG) or percutaneous coronary intervention (PCI) [8,9,15]. Similarly, for patients with two vessel disease and associated involvement of the proximal left anterior descending artery, studies suggest that there is benefit associated with undergoing revascularization [16,17]. The corollary to these benefits of revascularization is that undetected high-risk disease in a population is undesirable, as it may result in premature and/or preventable cardiac morbidity and mortality.

We present our results stratified by sex. Consistent with other work, there is less catheterization utilization in females [18-20]. In addition, the slope of the relationship between catheterization rate and high-risk cases detected differs between males and females indicating a lower high-risk case yield in females. This is consistent with the growing body of literature asserting that underlying disease and symptom presentation differs for females [19,20].

Although an indepth investigation of why this variation in the population catheterization rate exists across jurisdictions is outside the scope of this study, we do examine the patient characteristics across tertiles of catheterization utilization. The patient characteristics are very consistent across tertiles suggesting that differences are unlikely to be due to variations in underlying disease burden. Additionally, we examined the relationship stratified by indication for catheterization. Similar trends were observed as in the overall analysis indicating that a plateau in high risk detection has not been reached in the jurisdiction studied for either ACS or non-ACS subgroups.

If regional cardiac programs were to adopt a strategy of increasing cardiac catheterization rates, our study does not provide information about *how *higher population rates of cardiac catheterization ought to be achieved. Given current evidence though, it is likely to be more appropriate to increase the utilization of cardiac catheterization after acute coronary syndromes (ACS), since this approach is more consistent with existing clinical trials [15,21-23]. It is less certain whether it would be appropriate to increase catheterization rates in asymptomatic patients (or patients with stable angina) who have high risk features noted on non-invasive cardiac testing. Current work and guidelines, however, recommend increased use of non-invasive testing strategies and subsequent risk stratification which might lead to higher catheterization yield [24,25]. Future work should determine the optimal strategy for increasing detection of high-risk patients taking into account context specific factors such as the healthcare system, infrastructure, patient and provider preferences.

Implicit to the assertion of benefit associated with detection of high-risk disease is the assumption that intervention is of proven benefit. Of some concern, the clinical trials demonstrating benefit of CABG for patients with high-risk disease were conducted over 20 years ago, in highly selected patients with only stable angina (i.e. no ACS patients), and without the benefits of contemporary medical therapy [26]. And indeed, the results of the recent COURAGE trial underline the uncertainty surrounding the overall benefit of revascularization [27]. However, COURAGE did not include ACS patients and relatively few of the included patients had truly high-risk anatomy. Thus, this trial can not be considered as proof that revascularization is not beneficial. Rather, it does point to the persisting questions around the overall value of invasive cardiac procedures.

The economic considerations surrounding an increase in catheterization rates are also not explored by our analysis. A national strategy of intentionally seeking, and thus detecting, more individuals with high-risk disease would have the potential to put considerable pressure on existing personnel and infrastructure for revascularization procedures.

Our study has limitations. First, we examined population rates of cardiac catheterization without also assessing the appropriateness of procedures performed across the regions studied. However, it is unlikely that this significantly influences our study of the relationship between catheterization rates and yield of high-risk disease, because previous work has shown that geographic variability in catheterization rates is not significantly explained by inappropriate procedure use [28]. A second limitation is that we only age-adjusted and sex-stratified our catheterization rates. Our analysis would have been strengthened by further adjustment for the regional prevalence of CAD, but this information was not available to us. We suspect that this latter limitation does not systematically bias our results or undermine our main study finding. Lastly, our work does not consider the use of non-invasive testing. Its potential proliferation in coming years may affect both rates of catheterization and rates of high-risk yield. Future work will be needed to continuously examine the relationship between yield and catheterization rate as non-invasive imaging becomes more prevalent.

## Conclusions

We have demonstrated a consistent finding of linearly increasing yield of high-risk CAD as population rates of cardiac catheterization increase. This finding holds true across three Canadian jurisdictions, and over time, and its implications are relevant to the planning of cardiac procedure utilization not only in Canada, but also in countries with both lower and higher population rates of cardiac catheterization.

## Competing interests

The authors declare that they have no competing interests.

## Authors' contributions

FC, BM, PF, MG and WG contributed to the conception and design, analysis and interpretation of data. All authors contributed to the drafting and revision of the article for critically important intellectual content. All authors approved the final version of the manuscript.

## Pre-publication history

The pre-publication history for this paper can be accessed here:

http://www.biomedcentral.com/1472-6963/11/323/prepub
